# Lifelong Bilingualism and Literacy Skills Use Among Hispanic Adults

**DOI:** 10.1093/geroni/igaa057.1058

**Published:** 2020-12-16

**Authors:** Roberto Millar, Shalini Sahoo, Taka Yamashita, Phyllis Cummins

**Affiliations:** 1 Graduate Student, Baltimore, Maryland, United States; 2 University of Maryland, Baltimore, Elkridge, Maryland, United States; 3 University of Maryland, Baltimore, Maryland, United States; 4 Miami University, Oxford, Ohio, United States

## Abstract

Literacy skills use in everyday life is associated with social (e.g., civic participation) and economic (e.g., higher wages) benefits throughout the life course. Yet, the use of literacy skills may be lower among immigrants and those with limited language proficiency. No prior research examined associations between bilingualism in early life — a critical period for language acquisition — English literacy skills, and the use of literacy skills in later life of Hispanic adults. The objective of this study is to determine whether lifelong bilingualism (i.e., learning a second language in childhood and still understanding today) is associated with Hispanics’ everyday use of literacy skills later in life. Nationally representative data (n = 412) of Hispanics 35 years and older were obtained from the 2012/2014 Program for International Assessment of Adult Competencies (PIAAC). A series of logistic regression models were used to examine the associations between lifelong bilingualism and several measures of literacy skills use in everyday life. Results showed that lifelong bilingualism (vs. being monolingual) was negatively associated with the daily use of letters, notes, and e-mails (Odds Ratio = 0.426, p< 0.05), reading financial statements (Odds Ratio = 0.474, p< 0.05), and reading diagrams (Odds Ratio = 0.391, p< 0.05). Additionally, literacy was a consistent predictor of skill use. While bilingualism is generally beneficial for aging adults, our findings suggest that, among Hispanics, lifelong bilingualism is associated with the less frequent use of literacy skills later in life. We discuss the possible theoretical and practice implications of these findings.

